# Comparative safety of ustekinumab versus anti-TNF therapy during pregnancy in patients with inflammatory bowel disease: A systematic review and meta-analysis

**DOI:** 10.1097/MD.0000000000044393

**Published:** 2025-09-12

**Authors:** Ali Emre Bardak, Humza Saeed, Gizem Teker, Sonia Friedman, Saqr Alsakarneh, Stefan Mitev

**Affiliations:** a Department of Internal Medicine, Boston Medical Center – Brighton, Boston, MA; b Rawalpindi Medical University, Rawalpindi, Pakistan; c Istanbul University, Istanbul Medical Faculty, Istanbul, Turkey; d Division of Gastroenterology, Tufts University School of Medicine, Boston, MA; e Department of Internal Medicine, University of Missouri–Kansas City, Kansas City, MO; f Gastroenterology Clinic, University Hospital St Ivan Rilski, Sofia, Bulgaria.

**Keywords:** anti-TNF, Crohn disease, inflammatory bowel disease, pregnancy outcomes, ulcerative colitis, ustekinumab

## Abstract

**Background:**

Inflammatory bowel disease (IBD) frequently affects women of reproductive age. While the safety of anti–tumor necrosis factor (TNF) agents during pregnancy is well established, data on the safety of ustekinumab remain limited. We aimed to compare the safety of ustekinumab versus anti-TNF therapy in pregnant patients with IBD in terms of pregnancy outcomes.

**Methods:**

We systematically searched PubMed, Embase, and Cochrane databases. Studies comparing ustekinumab and anti-TNF agents and reporting key pregnancy outcomes were included. Pooled analyses were performed using random-effects models.

**Results:**

Four studies, encompassing 3308 pregnancies (592 ustekinumab, 2716 anti-TNF) were included. The majority of patients (2914; 88.2%) had Crohn disease, and the median disease duration ranged from 6.5 to 14 years. There was no significant difference between ustekinumab and anti-TNF therapy in major pregnancy outcomes, including live birth rates (67.2% vs 67.7%; odds ratio [OR] = 0.73, 95% confidence interval [CI] = 0.39–1.37), spontaneous abortion rates (5.9% vs 4.2%; OR = 1.51, 95% CI = 0.74–3.36), preterm delivery rates (6.6% vs 7.4%; OR = 0.50, 95% CI = 0.15–1.61), low birth weight rates (4.6% vs 7.1%; OR = 0.68, 95% CI = 0.23–1.98), and cesarean section rates (30.0% vs 30.1%; OR = 1.11, 95% CI = 0.85–1.45).

**Conclusion:**

Ustekinumab appears comparable to anti-TNF agents regarding major pregnancy outcomes in pregnant patients with IBD, suggesting its potential safety during pregnancy.

## 1. Introduction

The incidence of inflammatory bowel disease (IBD), encompassing Crohn disease and ulcerative colitis, peaks in the third decade of life,^[1,2]^ corresponding to the age range with the highest birth rates.^[3,4]^ Active disease during conception and pregnancy is associated with adverse fetal and maternal outcomes,^[5–7]^ making effective disease control imperative during this period. Consequently, biologic agents are increasingly used in pregnancy to control disease activity effectively.

Anti-tumor necrosis factor (anti-TNF) agents have been extensively investigated in the context of pregnancy and their safety profile is well established.^[8–12]^ In contrast, despite studies published in recent years,^[13–19]^ data on the safety of the use of ustekinumab during pregnancy remain limited due to the small number of studies, their small sample sizes and low statistical power.^[8,12,20,21]^ This limitation is largely attributable to the initially cautious approach toward ustekinumab use during pregnancy. Early observational studies included small cohorts and lacked a comperative group. As these studies and expert opinions provided more support for the use if ustekinumab during pregnancy, there has been an increase in the number of patients receiving ustekinumab during pregnancy, accompanied by an increase in both number of observational studies and their sample sizes over the past few years.^[12,21]^

Previous meta-analyses have either not reported the safety of ustekinumab use during pregnancy due to insufficient data or have presented the already limited data as a single-arm analysis because of the lack of comperative studies.^[11,22,23]^ When considering that disease activity could independently impact the pregnancy outcomes, incorporating a control arm helps reduce the confounding effects of underlying disease activity and other potential confounders. Given the consensus on their safety profile, and their broad adoption in clinical practice, anti-TNF agents could serve as a comparator in studies assessing newer therapies. In our meta-analysis, we aimed to compare ustekinumab versus anti-TNF therapy, focusing on pregnancy outcomes.

## 2. Materials and methods

This systematic review and meta-analysis was designed and conducted in line with the Cochrane Collaboration Handbook for systematic reviews of interventions.^[24]^ Reporting adhered to the preferred reporting items for systematic reviews and meta-analyses guidelines.^[25]^ The protocol was registered in the international prospective register of systematic reviews under registration number CRD42024612273.

### 2.1. Eligibility criteria

Studies meeting the following eligibility criteria were included: comparative observational studies (retrospective or prospective), involving pregnant patients diagnosed with IBD; including both a group exposed to ustekinumab and a group exposed to anti-TNF therapy; and reporting at least one outcome of interest. Studies were excluded if they: did not include both a group exposed to ustekinumab and a group exposed to anti-TNF therapy; involved overlapping patient populations; or failed to report maternal and/or fetal outcomes. There were no restrictions on publication date, language, or duration of follow-up.

### 2.2. Search strategy, study selection, and data extraction

PubMed, Embase, and Cochrane databases were systematically searched from inception to November 9, 2024. Following terms were used in the search strategy: “inflammatory bowel disease,” “IBD,” “Crohn,” “CD,” “ulcerative colitis,” “UC,” “biologic,” “ustekinumab,” “interleukin,” “IL-12,” “IL-23,” “anti-TNF,” “tumor necrosis factor inhibitor,” “infliximab,” “adalimumab,” “golimumab,” “certolizumab,” “pregnancy,” “pregnant,” “gestation,” “birth,” “prepartum,” “postpartum,” “infant,” “neonatal,” “breastfeeding,” “lactation.” The complete search strategy is presented in the Appendix, Supplemental Digital Content, https://links.lww.com/MD/P917. The reference lists of the included studies were reviewed to identify additional eligible studies.

Data were extracted for the following outcomes: live birth; spontaneous abortion; preterm delivery; low birth weight; and cesarean section.

Study selection and data extraction were performed independently by AEB and SM. Disagreements were resolved by consensus among the authors.

### 2.3. Definitions

Outcome and exposure definitions were accepted as provided in the individual studies. Specific details regarding how each study defined the exposure to anti-TNF therapy or ustekinumab during pregnancy and the outcomes of interest are presented in Table S1 (Supplemental Digital Content, https://links.lww.com/MD/P917) in the Appendix.

### 2.4. Quality assessment

The risk of bias was assessed using the Newcastle–Ottawa scale.^[26]^ This assessment was performed independently by SM and HS, with disagreements resolved by consensus.

### 2.5. Statistical analysis

Binary outcomes were compared using pooled odds ratios (ORs) with 95% confidence intervals (CIs), and *P*-values <.05 were considered statistically significant. Heterogeneity was evaluated using Cochran *Q* test and *I*² statistics. For Cochran *Q* test, *P*-values <.10 were considered indicative of significant heterogeneity. For the *I*² statistics, heterogeneity was categorized as low (≤25%), moderate (26%–50%), substantial (51%–75%), or considerable (>75%). The DerSimonian and Laird random-effects model was used for all outcomes.^[27]^ All statistical analyses were performed using Review Manager 5.4 (Nordic Cochrane Centre, The Cochrane Collaboration, Copenhagen, Denmark).

## 3. Results

### 3.1. Study selection and baseline characteristics

**Figure 1.**
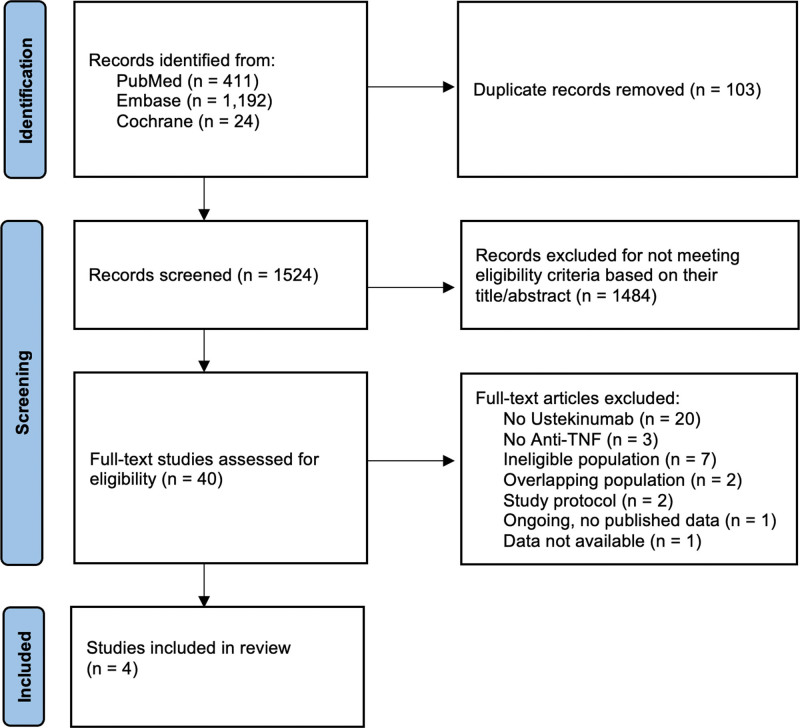
Preferred reporting items for systematic reviews and meta-analyses flow diagram of study screening and selection.

The initial search using the aforementioned search strategy yielded 1627 studies (Fig. 1). After excluding duplicate reports and studies that did not meet the eligibility criteria based on their titles or abstracts, 40 studies remained for full-text review. Two studies were excluded due to overlapping patient populations.^[6,13]^ The study by Mahadevan et al,^[6]^ which presented earlier results from the Pregnancy Inflammatory Bowel Disease and Neonatal Outcomes registry, overlapped with the continuation study by Chugh et al.^[16]^ Additionally, the study by Wils et al,^[13]^ which included data exclusively from centers in France, overlapped with the study by Meyer et al,^[17]^ as the latter encompassed all pregnancies in France between 2014 and 2021, including the population covered by Wils et al. Both studies were excluded to avoid duplication. Consequently, 4 studies were included in the meta-analysis.^[14–17]^ Of these, 3 were observational prospective studies,^[14–16]^ and 1 was an observational retrospective study.^[17]^

The general characteristics of the studies included in the meta-analysis are presented in Table 1. A total of 3308 pregnancies were included, of whom 592 (17.9%) were exposed to ustekinumab and 2716 (82.1%) were exposed to anti-TNF therapy. The majority of patients (2914; 88.2%) had Crohn disease, and median disease duration ranged from 6.5 to 14 years.

**Table 1:** Baseline characteristics of patients in included studies in the meta-analysis.

Study	Avni-Biron 2022^[14]^	Mitrova 2022^[15]^	Chugh 2024^[16]^	Meyer 2025^[17]^
Number of patients, n (%)				
Ustekinumab	27 (34.2)	54 (37.5)	47 (6.1)	464 (20.0)
Anti-TNF	52 (65.8)	90 (62.5)	718 (93.9)	1856 (80.0)
Age (yr)				
Ustekinumab	31.26 ± 5.92*	30 (27–34)†	33.0 ± 4.2*	30 (26–33)†
Anti-TNF	29.54 ± 4.00*	29 (25–33)†	31.9 ± 4.6*	30 (27–34)†
Gravidity				
Ustekinumab	2 (1.00–3.00)†	35 (64.8)‡	1.9 ± 1.0*	181 (39.0)‡
Anti-TNF	2 (1.25–4.00)†	59 (65.6)‡	2.1 ± 1.2*	716 (38.6)‡
Crohn disease, n (%)				
Ustekinumab	25 (92.6)	51 (94.4)	39 (83.0)	429 (92.5)
Anti-TNF	51 (98.1)	70 (77.8)	535 (74.9)	1714 (92.3)
Ulcerative colitis, n (%)				
Ustekinumab	1 (3.7)	3 (5.6)	8 (17.0)	35 (7.5)
Anti-TNF	1 (1.9)	20 (22.2)	168 (23.5)	142 (7.7)
Disease duration (yr)				
Ustekinumab	11 (8.00–15.00)†	13 (8–18)†	14.1 (9.7–17.3)†	8.6 (5.4–12.3)†
Anti-TNF	7 (4.00–12.00)†	6.5 (4–11)†	8.8 (4.9–13.5)†	8.6 (5.2–11.6)†
Concomitant thiopurine use, n (%)				
Ustekinumab	9 (33.3)	15 (27.8)	0 (0.0)	51 (11.0)
Anti-TNF	20 (39.2)	31 (34.3)	0 (0.0)	209 (11.3)

Anti-TNF = anti-tumor necrosis factor.

* Values are presented as mean ± standard deviation.

† Median (interquartile range)

‡ First pregnancy number (percentage).

### 3.2. Drug exposure

The Avni-Biron, Meyer, and Chugh studies considered exposure for ustekinumab as occurring 3 months prior to conception or during pregnancy, whereas the Mitrova study used a 2-month period. As for anti-TNF agents, exposure was defined as within 3 months prior to conception or during pregnancy in the Avni-Biron and Chugh studies, within 2 months in the Meyer study, and was not reported in the Mitrova study (Table S1, Supplemental Digital Content, https://links.lww.com/MD/P917).^[14–17]^

In the Avni-Biron study, patients receiving ustekinumab had been on therapy for a median of 12 months (interquartile range [IQR] = 5.75–18.25) before pregnancy, while this was 16.5 months (IQR = 6.0–36.0) for patients on anti-TNF agents, with the majority receiving adalimumab (65.3%), followed by infliximab (30.7%), and certolizumab (5.7%). Most common dose interval of ustekinumab was every 8 weeks (70%), followed by every 4 weeks (26%) and every 12 weeks (4%). Nearly all ustekinumab (92.5%) and anti-TNF (90.3%) treated patients continued therapy throughout the third trimester.^[14]^ In the Mitrova study, the median duration of drug use was 12.5 months (IQR = 5–21) for ustekinumab and 30 months (IQR = 11–55) for anti-TNF therapy. Among patients receiving anti-TNF therapy, 71.1% received infliximab and 29.9% received adalimumab. 30.2% of patients received ustekinumab every 4 to 6 weeks, while the remaining 69.8% followed a standard interval regimen. The final doses of drugs were administered at a median gestational week of 33 (range 18–38) in the ustekinumab group and 30 (range 22–39) in the anti-TNF group. 86.0% of the patients continued ustekinumab through the third trimester, while 11.6% discontinued it in the second trimester.^[15]^ In the Meyer study, ustekinumab was administered in 97.7% of pregnancies during the first trimester, 72.9% in the second, and 40.8% in the third. In comparison, anti-TNF agents were administered in 97.8%, 85.4%, and 66.3% of pregnancies during the first, second, and third trimesters, respectively.^[17]^ Finally, the Chugh study did not report specific dosing intervals or durations for ustekinumab or anti-TNF agents.^[16]^

### 3.3. Disease activity

In the Avni-Biron study, IBD was clinically active in the ustekinumab group in 11.1% of patients in the first trimester, 3.7% in the second, and 3.7% in the third, compared with 15.4%, 5.8%, and 3.8%, respectively, in the anti-TNF group.^[14]^ In the Mitrova study, disease activity was assessed using the physician global assessment and fecal calprotectin levels, indicating active disease in 16.7% of ustekinumab-treated and 10.0% of anti-TNF-treated pregnancies by physician global assessment, and in 42.2% versus 44.9%, respectively, by fecal calprotectin.^[15]^ The Meyer study did not report numerical measures of disease activity for either group. In terms of propensity score matching for the disease activity, both groups were matched using surrogate indicators of disease activity, such as IBD-related hospitalization and corticosteroid use in the 6 months preceding pregnancy.^[17]^ In the Chugh study, disease activity was assessed using the Harvey–Bradshaw Index for Crohn disease and the Simple Clinical Colitis Activity Index for ulcerative colitis, with the highest reported score serving as the representative disease activity for each pregnancy. 10.9% had active disease in the ustekinumab group, compared with 14.8% in the anti-TNF.^[16]^

### 3.4. Pooled analysis of pregnancy outcomes

**Figure 2.**
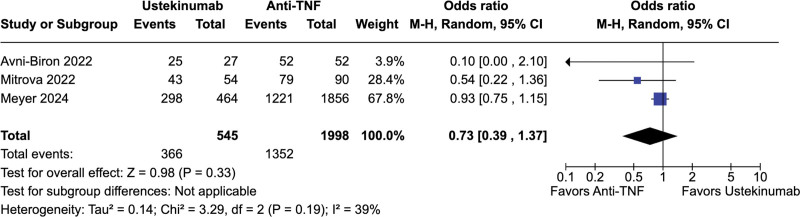
Forest plot of studies comparing live births in ustekinumab-exposed and anti-TNF-exposed pregnancies. anti-TNF = anti-tumor necrosis factor, CI = confidence interval, M–H = Mantel–Haenszel method.

**Figure 3.**
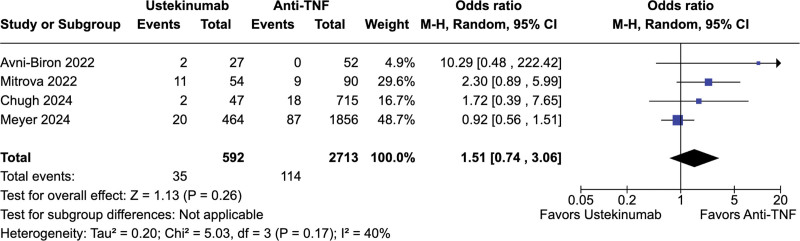
Forest plot of studies comparing spontaneous abortions in ustekinumab-exposed and anti-TNF-exposed pregnancies. anti-TNF = anti-tumor necrosis factor, CI = confidence interval, M–H = Mantel–Haenszel method.

**Figure 4.**
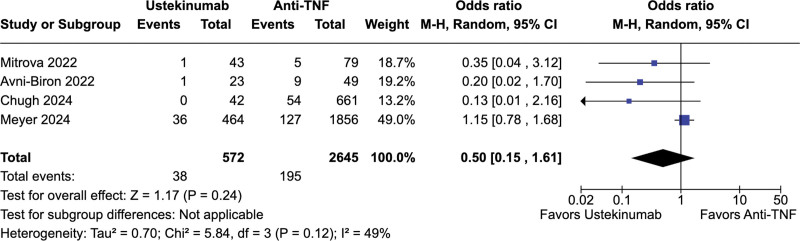
Forest plot of studies comparing preterm deliveries in ustekinumab-exposed and anti-TNF-exposed pregnancies. anti-TNF = anti-tumor necrosis factor, CI = confidence interval, M–H = Mantel–Haenszel method.

**Figure 5.**
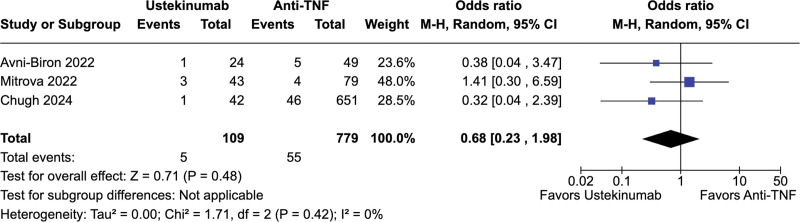
Forest plot of studies comparing low birth weight in ustekinumab-exposed and anti-TNF-exposed pregnancies. anti-TNF = anti-tumor necrosis factor, CI = confidence interval, M–H = Mantel–Haenszel method.

**Figure 6.**
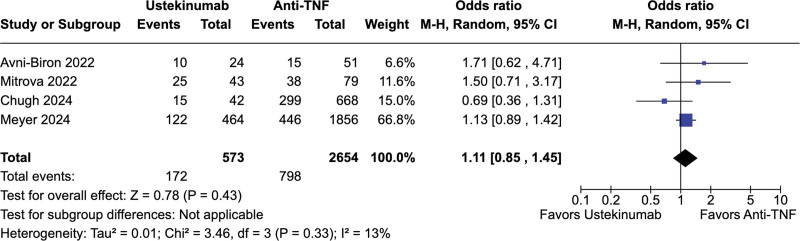
Forest plot of studies comparing cesarean sections in ustekinumab-exposed and anti-TNF-exposed pregnancies. anti-TNF = anti-tumor necrosis factor, CI = confidence interval, M–H = Mantel–Haenszel method.

No significant difference was observed between ustekinumab-exposed and anti-TNF-exposed pregnancies regarding live births (67.2% vs 67.7%; OR = 0.73; 95% CI = 0.39–1.37; *P* = .33; *I*^2^ = 39%; Fig. 2), spontaneous abortion (5.9% vs 4.2%; OR = 1.51; 95% CI = 0.74–3.36; *P* = .26; *I*^2^ = 40%; Fig. 3), preterm delivery (6.6% vs 7.4%; OR = 0.50; 95% CI = 0.15–1.61; *P* = .24; *I*^2^ = 49%; Fig. 4), low birth weight (4.6% vs 7.1%; OR = 0.68; 95% CI = 0.23–1.98; *P* = .48; *I*^2^ = 0%; Fig. 5), and cesarean delivery (30.0% vs 30.1%; OR = 1.11; 95% CI = 0.85–1.45; *P* = .43; *I*^2^ = 3%; Fig. 6).

### 3.5. Quality assessment

Risk of bias assessment was performed for each study using the Newcastle–Ottawa Scale,^[26]^ which indicated a low risk of bias for all 4 studies in Table S2 (Supplemental Digital Content, https://links.lww.com/MD/P917) in the Appendix.

## 4. Discussion

In this systematic review and meta-analysis, the safety of ustekinumab and anti-TNF therapy in pregnant patients with IBD was compared in terms of pregnancy outcomes. The pooled analysis did not show any significant difference between the 2 therapies for live birth, spontaneous abortion, preterm delivery, low birth weight, and cesarean section rates.

The majority of the patients (88.2%) in the meta-analysis had Crohn disease. Although this proportion might appear disproportionately high, it reflects the fact that ustekinumab is more commonly used for Crohn disease than for ulcerative colitis. Similar distributions have been observed in meta-analyses assessing the safety of ustekinumab in IBD, and in this context, the rate in our study is reassuring.^[23,28]^

Disease activity during pregnancy is a critical determinant of maternal and fetal outcomes in IBD. A meta-analysis by Kim et al demonstrated significantly higher odds of preterm birth (OR = 2.42), low birth weight (OR = 3.81), and spontaneous abortion (OR = 1.87) among pregnant women with active disease compared to those without active disease.^[5]^ Similarly, Mahadevan et al showed that higher disease activity was associated with spontaneous abortion (hazard ratio 3.41) and preterm birth (OR = 1.73).^[6]^ Therefore, disease activity may be a confounder in the assessment of these outcomes. Across the included studies, in the Avni-Biron study, ustekinumab-treated patients had a significantly higher rate of active disease in the first and third trimesters compared with anti-TNF controls, yet no significant differences in new flares, corticosteroid use, or biomarker levels were noted.^[14]^ The Mitrova study similarly reported a slightly higher percentage of clinically active disease in the ustekinumab group, whereas fecal calprotectin-based assessments were comparable between the 2 therapies.^[15]^ The Chugh study observed more active disease in the anti-TNF group than in the ustekinumab group,14.8% versus 10.9%.^[16]^ Finally, the Meyer study used propensity score matching for the disease activity and severity, and they also conducted subgroup analyses comparing both therapies by stratifying patients into inactive and active disease groups.^[17]^ In both subgroups, there were no significant differences between the 2 therapies for any of the evaluated outcomes, including preterm birth, birth weight, and type of delivery.

Another important aspect to consider is that ustekinumab is frequently used in patients with inadequate response, treatment failure, drug resistance, or adverse effects to other biologic therapies, including anti-TNF agents.^[29–32]^ For instance, in the Avni-Biron study, among patients receiving ustekinumab, 77.8% had previously experienced failure with infliximab, 92.6% with adalimumab, and 48.1% with vedolizumab.^[14]^ Similarly, the Mitrova study reported that women receiving ustekinumab were younger at diagnosis, had longer disease duration, and had more intestinal surgeries compared to those on anti-TNF agents.^[15]^ In this context, the use of ustekinumab also serves as an indicator of more refractory IBD, and controlling disease activity can be more challenging for those on ustekinumab compared to those on anti-TNF agents.

Placental transfer of ustekinumab and anti-TNF agents, except certolizumab, starts in the second trimester and occurs predominantly in the third trimester.^[33,34]^ This is primarily driven by the exponential increase in fragment crystallizable receptor expression in the placenta, facilitating the active transport of immunoglobulin G antibodies, including ustekinumab and anti-TNF agents, to the fetal circulation.^[18,35,36]^ Maternal levels of ustekinumab remain stable throughout pregnancy, and infant levels at delivery are higher than maternal levels.^[15,16,33]^ The median infant-maternal ratio of ustekinumab at delivery has been reported as 1.67,^[15]^ 1.46,^[16]^ 2.18,^[19]^ and 1.79.^[33]^ Additionally, it is worth noting that maternal and infant ustekinumab levels at delivery are strongly correlated (*R* = 0.75; *P* = .001), and shorter intervals between the final dose and delivery result in higher ustekinumab levels in infants (*r* = −0.65; *P* = .006), potentially contributing to the minor variability across studies.^[33]^

Ustekinumab, infliximab, and adalimumab exhibit similar placental transfer profiles, resulting in comparable fetal exposure,^[37]^ while certolizumab has minimal placental transfer due to its lack of an fragment crystallizable region.^[37,38]^ No studies to date have measured fetal ustekinumab levels during early and mid-pregnancy, likely due to practical and ethical challenges.

Recommendations for the use of ustekinumab during pregnancy have evolved as new studies and data have been published. In the 2019 IBD in pregnancy Clinical Care Pathway Report from the American Gastroenterological Association IBD Parenthood Project Working group, it was recommended to discontinue ustekinumab 6 to 10 weeks before the expected due date of pregnancy.^[8]^ Although biologic agents are generally used at lower doses in rheumatologic diseases than in IBD, the 2020 American College of Rheumatology Guideline for the Management of reproductive Health in Rheumatic and Musculoskeletal Diseases’ approach was still cautious, recommending the continuation of ustekinumab while trying to conceive but its discontinuation after conception.^[39–42]^ The 2023 European Crohn and Colitis Guidelines on Sexuality, Fertility, pregnancy, and Lactation classified ustekinumab as low risk during pregnancy but with limited data, recommending close monitoring of patients due to the limited evidence.^[12]^ Similarly, the 2024 Updates on the Management of IBD from Periconception to Pregnancy and Lactation suggested that, based on limited available data, the use of ustekinumab is safe during the periconceptional period, pregnancy, and lactation, with no alarming safety signals identified.^[21]^ With recent studies, data supporting the use of ustekinumab during pregnancy continue to expand.

This meta-analysis is the first to investigate the safety of ustekinumab for major pregnancy outcomes by comparing it with anti-TNF therapy, yet it has several limitations. First, it included a relatively small number of studies, which reduced both the overall sample size and the statistical power of the analyses. Also, it limited our ability to perform subgroup analyses.

Second, owing to the nature of this topic, no randomized controlled trials have been conducted; thus, only observational studies were available, potentially introducing bias. Third, not all included studies matched treatment groups according to disease activity or other baseline characteristics, such as age, parity, or immunomodulator use, which may have introduced additional confounding. The Meyer study used propensity score matching, and in the Avni-Biron study, the ustekinumab group was matched based on age, body mass index, and parity,^[14,17]^ whereas the Mitrova and Chugh studies did not implement any matching method.^[15,16]^ Finally, the meta-analysis revealed moderate heterogeneity for live birth, spontaneous abortion, and preterm delivery.

## 5. Conclusions

No significant differences in major pregnancy outcomes were found between ustekinumab-exposed and anti-TNF-exposed pregnancies. These findings suggest the use of ustekinumab during pregnancy is comparable with anti-TNF therapy and can be considered safe. Nevertheless, it should be acknowledged that the data are still limited, and further studies are needed.

## Acknowledgments

The authors have no additional contributions or external support to acknowledge for this work.

## Author contributions

**Conceptualization:** Ali Emre Bardak, Gizem Teker.

**Data curation:** Ali Emre Bardak, Humza Saeed, Saqr Alsakarneh, Stefan Mitev.

**Formal analysis:** Ali Emre Bardak, Stefan Mitev.

**Investigation:** Ali Emre Bardak.

**Methodology:** Ali Emre Bardak, Stefan Mitev.

**Visualization:** Ali Emre Bardak.

**Writing – original draft:** Ali Emre Bardak.

**Writing – review & editing:** Ali Emre Bardak, Sonia Friedman, Stefan Mitev.

## Supplementary Material


