# Supplementary material for: Comparative safety of ustekinumab versus anti-TNF therapy during pregnancy in patients with inflammatory bowel disease: A systematic review and meta-analysis
Source: Medicine (Baltimore). 2025 Sep 12;104(37):e44393. doi: 10.1097/MD.0000000000044393 (PMC12440402; doi:10.1097/MD.0000000000044393)
Supplement: Supplementary file 1 [file medi-104-e44393-s001.docx]

**Supplementary Table 1.** Outcome and exposure definitions across included studies in the meta-analysis.

| Study | Avni-Biron 2022 | Mitrova 2022 | Meyer 2024 | Chugh 2024 |
| --- | --- | --- | --- | --- |
| Ustekinumab exposure | All selected patients were treated with their medication for at least 3 months prior to conception. All patients continued Ustekinumab therapy during pregnancy. | All selected patient were exposed to Ustekinumab within 2 months prior to conception or during pregnancy. | All selected patient were exposed to Ustekinumab within 3 months prior to conception or during pregnancy. | All selected patient were exposed to Ustekinumab within the 3 months prior to last menstrual period or during pregnancy. |
| Anti-TNF agent exposure | All selected patients were treated with their medication for at least 3 months prior to conception. All patients were on anti-TNF therapy at conception. | Pregnancies, that are exposed to anti-TNF. No specific definition of exposure. | All selected patient were exposed to anti-TNF within 2 months prior to conception or during pregnancy. | All selected patient were exposed to anti-TNF within the 3 months prior to last menstrual period or during pregnancy. |
| Anti-TNF agents | IFX, ADA, CLZ | IFX, ADA | IFX, ADA, GOL | IFX, ADA, GOL, CLZ |
| OUTCOMES |  |  |  |  |
| Live birth | Not defined | Not defined | Not defined | Not reported |
| Spontaneous Abortion | A pregnancy loss before 20 gestational weeks | Not defined | Not defined | Not defined |
| Preterm birth | Delivery before 37+0 gestational weeks | Not defined | Delivery before 37 gestational weeks. Very preterm birth, which is defined as delivery before 32 gestational weeks is included in preterm birth in our meta-analysis. | Delivery before 37 gestational weeks |
| C-section | Not defined | Not defined | Not defined | Not defined |
| Low birth weight | Birth weight < 2500 grams | Birth weight < 2500 grams | Not reported | Birth weight < 2500 grams |
|  |  |  |  |  |

**Supplementary Table 2.** Risk of bias assessment of observational studies using Newcastle-Ottawa Scale. The following sections in the table were rated per study and the bias was assessed as: low bias risk (7-9 points), moderate bias risk (4-6 points) and high bias risk (0-3 points).

|  | Selection | | | | Comparability | Outcome | | |  |
| --- | --- | --- | --- | --- | --- | --- | --- | --- | --- |
| Study | Representative of exposed cohorts | Selection of the non-exposed cohort | Ascertainment of exposure | Demonstration that outcome of interest was not present at the start of study | Comparability of cohort on the basis of design or analysis | Assessment of outcome | Was follow-up long enough for the outcomes to occur | Adequacy of follow-up of cohorts | Total score |
| Avni-Biron 2022 | 1 | 1 | 1 | 1 | 2 | 1 | 1 | 1 | 9 |
| Mitrova 2022 | 1 | 1 | 1 | 1 | 0 | 1 | 1 | 1 | 7 |
| Chugh 2024 | 1 | 1 | 1 | 1 | 0 | 1 | 1 | 1 | 7 |
| Meyer 2024 | 1 | 1 | 1 | 1 | 2 | 1 | 1 | 1 | 9 |

**Search Strategy Used in PubMed, Embase, and Cochrane Databases**

(inflammatory bowel disease OR IBD OR Crohn OR CD OR ulcerative colitis OR UC)

AND (prepartum OR pregnancy OR pregnant OR gestation OR birth OR breastfeeding OR lactation OR postpartum OR infant OR neonatal)

AND (biologic OR ustekinumab OR interleukin OR IL-12 OR IL-23)

AND (anti-TNF OR tumor necrosis factor inhibitor OR infliximab OR adalimumab OR golimumab OR certolizumab)
